# How precise are oral splints for frameless stereotaxy in guided ear, nose, throat, and maxillofacial surgery: a cadaver study

**DOI:** 10.1186/s41747-021-00223-3

**Published:** 2021-06-30

**Authors:** Manfred Nilius, Minou Hélène Nilius

**Affiliations:** 1 NILIUSKLINIK Dortmund, Londoner Bogen 6, D-44269 Dortmund, Germany; 2 Technische Universität Dresden, Dresden, Germany

**Keywords:** Neuronavigation, Fiducial markers, Occlusal splints, Surgery (computer-assisted)

## Abstract

**Background:**

Computer-assisted surgery optimises accuracy and serves to improve precise surgical procedures. We validated oral splints with fiducial markers by testing them against rigid bone markers.

**Methods:**

We screwed twenty bone anchors as fiducial markers into different regions of a dried skull and measured the distances. After computed tomography (CT) scanning, the accuracy was evaluated by determining the markers’ position using frameless stereotaxy on a dry cadaver and indicated on the CT scan. We compared the accuracy of chairside fabricated oral splints to standard registration with bone markers immediately after fabrication and after a ten-time use. Accuracy was calculated as deviation (mean ± standard deviation). For statistical analysis, *t* test, Kruskal-Wallis, Tukey's, and various linear regression models, such as the Pearson's product–moment correlation coefficient, were used.

**Results:**

Oral splints showed an accuracy of 0.90 mm ± 0.27 for viscerocranium, 1.10 mm ± 0.39 for skull base, and 1.45 mm ± 0.59 for neurocranium. We found an accuracy of less than 2 mm for both splints for a distance of up to 152 mm. The accuracy persisted even after ten times removing and reattaching the splints.

**Conclusions:**

Oral splints offer a non-invasive indicator to improve the accuracy of image-guided surgery. The precision is dependent on the distance to the target. Up to 150-mm distance, a precision of fewer than 2 mm is possible. Dental splints provide sufficient accuracy than bone markers and may opt for higher precision combined with other non-invasive registration methods.

## Key points

- Oral splints are easy to prepare chairside for acutely traumatised patients.
- Computer-assisted procedures can guide surgery after initial diagnosis thanks to the use of oral splints.
- Evaluation of treatment after tumour resection or reconstruction is possible using oral splints.

## Background

Computer-assisted digital planning tools support medical diagnosis and treatment. They increase precision in operations and can be useful in anatomically complex regions that are difficult to access. It is necessary to ensure an identical position of the patient on the computer display before and during the operation. Marking procedures help correlate the patient's anatomy with the three-dimensional reconstructed anatomy [1–3]. Different factors influence the accuracy: the slice thickness of computed tomography (CT) or cone beam CT (CBCT) data, the reconstruction algorithms, and the internal accuracy of the tracking system used *in vivo* [4, 5].

Before introducing computer-assisted tools, intraoperative stereotactic surgery was used since the beginning of the last century, using a stereotactical frame attached to the patient's head. There are, however, disadvantages to these systems. Complex or elongated areas of operation lead to inaccuracies in the correlation. The mechanical arms can impede the therapist during the operation.

In addition to frame-based and mechanical procedures [6, 7], there are frameless stereotactical models based on the principle of satellite navigation. Standardised systems work with infrared light-emitting diodes. The navigated instrument's position can be determined. In 2012, Ledderhose et al. [7] reported accuracy of less than 1 mm for these systems. Several authors reported using titanium screws for navigation inserted in the skull before neurosurgical and maxillofacial operations [4, 8]. Titanium screws or plates already inserted in earlier operations may also be helpful for navigation. Watanabe et al. [9] even described the use of small holes (*e.g.*, obtained using trepans) to assist in navigation.

The use of anatomical landmarks like osseous tuberosities of the skeleton to correlate the patient's preoperative and perioperative position is possible. The use of teeth and the edges of fillings for navigation is also an option [10].

A special kind of correlation is surface matching. A preoperative scan of the patient's body surface performed by a scanner or video camera serves the surfaces' intraoperative calibration and guarantees their precise positioning [11, 12]. Scanning 15 to 40 defined or coincidental surface points is necessary. The overlay of the current video and the patient data three-dimensional reconstruction to pursue the patient's head's exact position was described by Ji et al. [13]. Inaccuracies occur when soft tissue is oedematous or traumatised, and operational intervention is delayed [14]. Some authors described the use of surgical navigation assisted by augmented reality [15–18]. Yao et al. [19] compared the accuracy of augmented reality technology and navigation.

There are advantages and disadvantages for frame-based, invasive, or non-invasive methods and different accuracies for the chosen technique. No procedure has become generally accepted as a standard due to many possible marking methods. For this reason, this study aimed to present an exact, easy-to-use marking mechanism to serve the positioning of the patient and orientation in surgery for oral and maxillofacial, ear, nose, throat, and neurosurgery with an accuracy of less than 1 mm.

## Methods

### CT scan and navigation system

The measurements were performed on a dry cadaver head (obtained from the Institute of Anatomy at the University of Freiburg), armed with twenty standardised bone markers in different head regions (Howmedica-Leibinger, Freiburg, Germany). Axial spiral CT scans were obtained (Somatom Sensation 64, Siemens, Germany) (kV 120; effective mAs 300; rotation time 1.0 s.; slice collimation 1.2 mm; slice width 3.0 mm; feed/rotation 19.2 mm; pitch factor 0.8; reconstruction increment 1.0 mm; Kernel H60s; CTDI Vol 59.4 mGy; effective dose: 2.85 mSv) and transferred to the Surgical Tool Navigator System, software STP4 (Carl Zeiss, Jena, Germany).

### Oral splints

**Fig. 1:**
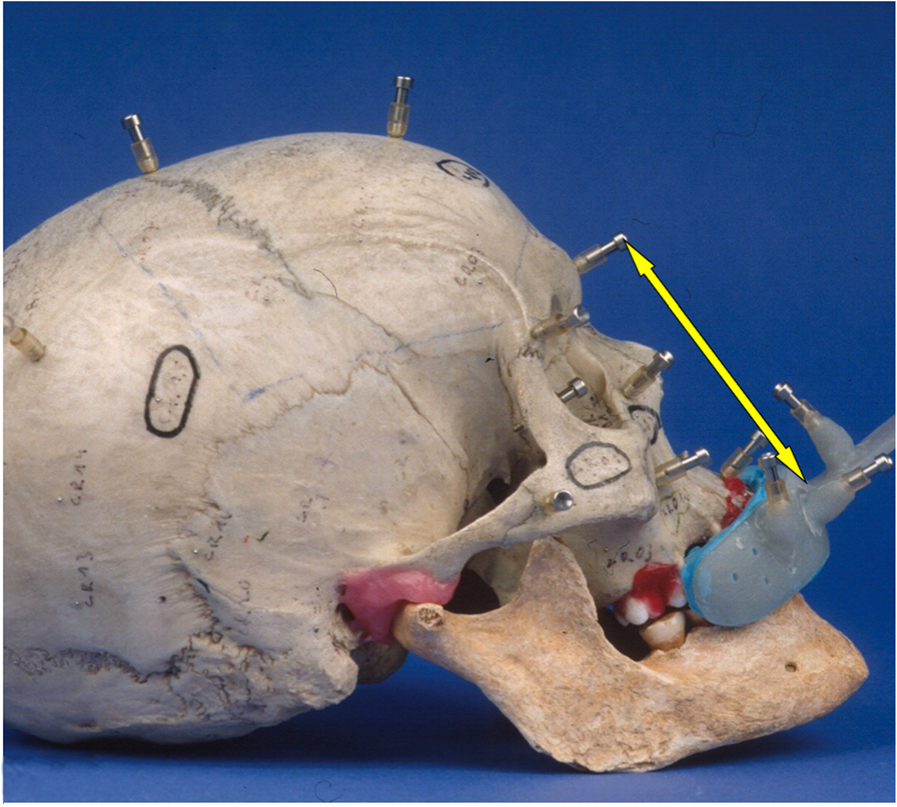
Part of the skull's viscerocranium (detail) with a vestibular splint (VestS) and bone markers. The arrow shows the distance between the centre of the VestS and the bone marker at the glabella

We prepared maxillary splints (MaxS) consisting of a perforated dental impression tray of the size BO0 to BOIII (Vita, Bad Saeckingen, Germany) in combination with commercial A-silicones such as Flexitime Monophase (Heraeus-Kultaer, Dormagen, Germany) or Dimension Bite 60 Sec (Espe, Seefeld, Germany) (Fig. 1). Prefabricated connector sockets (Howmedica-Leibinger, Freiburg, Germany) were attached to the trays’ vestibular surfaces. These served to plug on standardised navigation markers. The base plugs' adjustment took place in *x*-, *y*-, and *z*-directions, defined precisely with the vectors, maximal deflection in the three space levels. A plastic handle was fixed to the anterior part of the external tray surface to carry a subnasal marker (Figs. 3 and 4).

A vestibular splint (VestS) consisted of synthetic material hardened by light (Voco, Cuxhaven, Germany) in the shape of a vestibular pacifier. We polymerised one nonstandard grasp and four base items at the labial site to accommodate fiducials polymethylmethacrylate (Ortocryl Dentaurum J. P. Winkelstroeter KG, Ispringen, Germany). The adjustment of the base items took place according to the orientation of the MaxS. Impression was performed as monoblock in intermaxillary intercuspidation as described by Singer-Sosnowski [20]. We used the same A-silicones for splint production as for MaxS (Figs. 1 and 5).

### Metric accuracy test

**Fig. 2:**
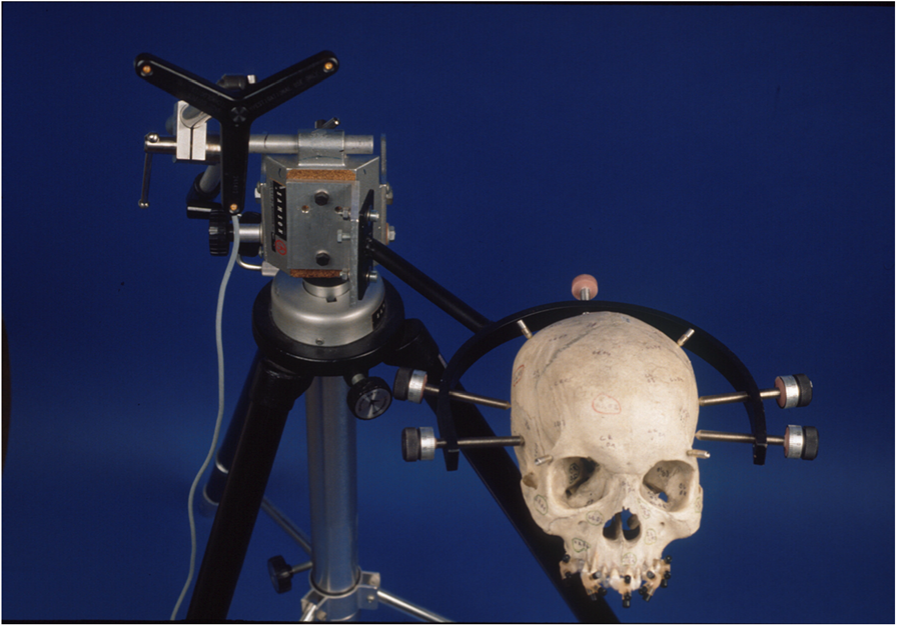
Skull in a modified Mayfield clamp fixed with five conical screws. Static fastening of the skull at a tripod and connection to a dynamic reference frame (DRF)

We screwed a total of twenty standardised bone markers in different head regions in addition to the fiducials fastened to the splints and fixed MaxS or VestS to a dry skull's teeth to examine metric accuracy (Fig. 2). The markers encircled different groups: neurocranium, skull base, and viscerocranium (Table 1). The splint centre was at the base of the splint in direct contact with the subnasal fiducial marker and continuation of the spoon grasp and the estimated occlusal plane (Figs. 1 and 2). The distance from the splint centre to the bone markers indicates the starting point (Table 2). We correlated the accuracy for MaxS and VestS to the distance of the head's determined bone marker (Table 3).

**Table 1:** Accuracy of two oral splints for guided surgery in different regions of the head

Region/ splint	MaxS (Mean ± SD)	VestS (Mean ± SD)
**Viscerocranium**	0.90 ± 0.27 mm	0.90 ± 0.53 mm
**Skull base**	1.10 ± 0.39 mm	1.20 ± 0.37 mm
**Neurocranium**	1.45 ± 0.59 mm	1.70 ± 0.76 mm

Accuracy of maxillary (MaxS) and vestibular splints (VestS) for the region: viscerocranium, skull base, and neurocranium in mm (mean ± standard deviation (SD)).

MaxS: chairside fabricated Maxillary splint, fixed on dentate maxilla

VestS: chairside fabricated Vestibular splint (pacifier), fixed as intermaxillary pacifier

**Table 2:** Assignment of 20 bone markers to three head regions (viscerocranium “V,” neurocranium “N,” and skull base “S”) and their distance to oral splints

Marker number	Localisation	Group	Distance from marker to splint’s median point (cm)
1	Hard palate	V	4.3
2	Subspinale processus	V	1.5
3	Right maxilla	V	4.6
4	Left zygomaticomaxillary suture	V	6.2
5	Right infraorbital rim	V	5.5
6	Right zygomatical anterior arch	V	6.6
7	Left lateral zygomatical arch	V	10.3
8	Right infraorbital zygomatic corpus	V/S	7.7
9	Right fronto zygomatico processus	N/S	10
10	Right orbital roof	N/S/V	7.7
11	Left lateral orbital roof	N/S/V	9.8
12	Glabella	N/S/V	7.5
13	Anterior frontal bone	N	14.4
14	Vortex	N	18.7
15	Lambda suture	N	22.5
16	Median occipital bone	N	24.5
17	Right parietal bone	N	21.2
18	Foramen magnum	N/S	9.3
19	Left parietal bone	H	15.2
20	Left mastoid	N/S	14.7

*V* Viscerocranium, *N* Neurocranium, *S* Skull base

The right column shows the distance from the splint’s centre point to the fiducial bone markers in cm. Points 8–12, 18, and 20 were assigned to several groups

**Table 3:** Precision of two different splints (MaxS and VestS) measured at twenty bone marker points. Group A was measured immediately after completion. Group B was reattached ten times and measured. Accuracy (Mean ± SD) as difference between position of the targets in the CT and the targets position using a navigation system

**a**					
**MARKER**	**A-GROUP**	**B-GROUP**	**A-GROUP**	**B-GROUP**	**A-GROUP**
	**MaxS 1 (A)**	**MaxS 1 (B)**	**MaxS 2 (A)**	**MaxS 2 (B)**	**MaxS 3 (A)**
**ISR**	0.28 ± 0.10	0.30 ± 0.09	0.34 ± 0.01	0.36 ± 0.12	0.38 ± 0.11
1	0.40 ± 0.10	0.44 ± 0.10	0.39 ± 0.08	0.49 ± 0.17	0.42 ± 0.07
2	0.59 ± 0.07	0.64 ± 0.10	0.72 ± 0.16	0.71 ± 0.08	0.59 ± 0.06
3	0.54 ± 0.05	0.67 ± 0.13	1.21 ± 0.12	1.10 ± 0.16	0.52 ± 0.08
4	0.57 ± 0.11	0.72 ± 0.11	1.29 ± 0.09	1.23 ± 0.20	0.78 ± 0.07
5	0.89 ± 0.11	1.00 ± 0.12	1.19 ± 0.11	1.19 ± 0.15	0.82 ± 0.07
6	0.89 ± 0.11	0.93 ± 0.22	1.42 ± 0.16	1.58 ± 0.23	0.74 ± 0.17
7	0.94 ± 0.10	1.04 ± 0.13	0.23 ± 0.09	0.46 ± 0.10	0.87 ± 0.10
8	0.94 ± 0.08	1.07 ± 0.14	1.29 ± 0.09	1.50 ± 0.21	0.70 ± 0.09
9	0.84 ± 0.05	0.94 ± 0.11	1.94 ± 0.11	2.13 ± 0.17	061 ± 0.06
10	0.94 ± 0.11	1.10 ± 0.19	1.81 ± 0.08	2.19 ± 0.25	1.42 ± 0.24
11	0.49 ± 0.09	0.79 ± 0.14	1.98 ± 0.12	2.20 ± 0.20	1.48 ± 0.08
12	0.99 ± 0.07	1.23 ± 0.22	1.98 ± 0.17	1.99 ± 0.26	1.87 ± 0.10
13	0.99 ± 0.09	1.11 ± 0.15	2.92 ± 0.13	2.96 ± 0.19	2.07 ± 0.28
14	1.54 ± 0.10	1.87 ± 0.16	4.60 ± 0.28	4.70 ± 0.47	4.37 ± 0.15
15	2.44 ± 0.13	2.57 ± 0.18	5.28 ± 0.26	5.47 ± 0.43	5.81 ± 0.15
16	2.13 ± 0.13	2.23 ± 0.15	7.42 ± 0.12	7.58 ± 0.25	8.51 ± 0.20
17	1.61 ± 0.09	1.59 ± 0.11	4.89 ± 0.13	4.93 ± 0.27	4.87 ± 0.15
18	1.13 ± 0.11	1.17 ± 0.15	1.14 ± 0.13	1.17 ± 0.20	1.63 ± 0.13
19	2.24 ± 0.18	2.24 ± 0.18	4.86 ± 0.15	4.84 ± 0.32	4.48 ± 0.32
20	2.03 ± 0.21	2.00 ± 0.13	3.71 ± 0.28	3.74 ± 0.26	3.70 ± 0.19
**b**					
**MARKER**	**B-GROUP**	**A-GROUP**	**B-GROUP**	**A-GROUP**	**B-GROUP**
	**MaxS 3 (B)**	**MaxS 4 (A)**	**MaxS 4 (B)**	**MaxS 5 (A)**	**MaxS 5 (B)**
**ISR**	0.34 ± 0,09	0.50 ± 0.04	0.45 ± 0.06	0.47 ± 0.04	0.51 ± 0.05
1	0.49 ± 0.12	0.69 ± 0.08	1.09 ± 0.18	0.58 ± 0.08	0.74 ± 0.18
2	0.66 ± 0.10	0.69 ± 0.09	0.74 ± 0.11	0.66 ± 0.09	0.81 ± 0.26
3	0.81 ± 0.13	0.51 ± 0.09	0.78 ± 0.14	0.94 ± 0.10	1.00 ± 0.22
4	0.81 ± 0.12	0.96 ± 0.10	0.89 ± 0.18	1.46 ± 0.21	1.59 ± 0.20
5	1.00 ± 0.16	0.58 ± 0.16	0.82 ± 0.30	1.09 ± 0.13	1.18 ± 0.17
6	0.98 ± 0.20	0.99 ± 0.11	0.99 ± 0.20	1.26 ± 0.15	1.29 ± 0.21
7	0.90 ± 0.17	1.01 ± 0.18	1.18 ± 0.19	1.47 ± 0.12	1.62 ± 0.15
8	0.87 ± 0.18	0.93 ± 0.11	1.10 ± 0.21	1.61 ± 0.09	1.90 ± 0.19
9	0.80 ± 0.19	1.42 ± 0.15	1.31 ± 0.31	1.97 ± 0.11	2.04 ± 0.17
10	1.51 ± 0.24	1.42 ± 0.13	1.37 ± 0.28	1.91 ± 0.14	2.26 ± 0.21
11	1.19 ± 0.31	0.84 ± 0.09	1.00 ± 0.18	2.00 ± 0.26	2.19 ± 0.28
12	1.67 ± 0.23	0.99 ± 0.08	1.16 ± 0.17	1.50 ± 0.11	1.56 ± 0.19
13	2.20 ± 0.50	1.12 ± 0.10	1.23 ± 0.13	1.41 ± 0.09	1.50 ± 0.12
14	4.00 ± 1.15	2.11 ± 0.16	2.12 ± 0.32	1.81 ± 0.27	2.04 ± 0.21
15	5.07 ± 1.32	3.48 ± 0.16	3.43 ± 0.72	4.99 ± 0.14	5.09 ± 0.29
16	5.68 ± 2.25	3.36 ± 0.30	3.44 ± 0.67	3.82 ± 0.29	4.37 ± 0.31
17	4.90 ± 0.73	2.01 ± 0.20	2.09 ± 0.41	4.51 ± 0.19	4.53 ± 0.21
18	1.78 ± 0.37	1.32 ± 0.13	1.32 ± 0.21	1.52 ± 0.13	1.69 ± 0.20
19	4.11 ± 0.80	1.79 ± 0.08	2.13 ± 0.25	4.42 ± 0.39	4.61 ± 0.39
20	3.82 ± 1.00	1.92 ± 0.14	1.97 ± 0.23	4.59 ± 0.15	4.54 ± 0.37
**c**					
**MARKER**	**A-GROUP**	**B-GROUP**	**A-GROUP**	**B-GROUP**	**A-GROUP**
	**VestS 6 (A)**	**VestS 6 (B)**	**VestS 7 (A)**	**VestS 7 (B)**	**VestS 8 (A)**
**ISR**	0.23 ± 0.03	0.37 ± 0.07	0.40 ± 0.04	0.39 ±0.13	0.50 ± 0.06
1	0.56 ± 0.07	0.74 ± 0.18	0.47 ± 0.07	0.69 ± 0.21	0.50 ± 0.07
2	0.57 ± 0.12	0.67 ± 0.12	0.61 ± 0.06	0.66 ± 0.16	0.56 ± 0.20
3	1.01 ± 0.11	1.03 ± 0.26	0.90 ± 0.10	0.91 ± 0.14	0.44 ± 0.05
4	1.27 ± 0.12	1.52 ± 0.18	1.36 ± 0.09	1.38 ± 0.15	0.52 ± 0.12
5	0.92 ± 0.07	1.06 ± 0.17	1.04 ± 0.09	1.09 ± 0.13	0.83 ± 0.09
6	1.33 ± 0.09	1.37 ± 0.19	1.18 ± 0.11	1.07 ± 0.16	0.82 ± 0.08
7	1.46 ± 0.05	1.60 ± 0.12	1.40 ± 0.07	1.52 ± 0.12	0.89 ± 0.08
8	1.68 ± 0.26	2.02 ± 0.29	1.50 ± 0.09	1.66 ± 0.24	0.70 ± 0.09
9	1.92 ± 0.15	2.08 ± 0.19	2.04 ± 0.14	2.02 ± 0.14	0.63 ± 0.05
10	1.93 ± 0.13	2.34 ± 0.15	1.90 ± 0.09	2.16 ± 0.30	1.52 ± 0.24
11	1.92 ± 0.13	2.41 ± 0.21	1.89 ± 0.09	1.96 ± 0.15	1.59 ± 0.08
12	1.08 ± 0.11	1.33 ± 0.17	1.07 ± 0.10	1.09 ± 0.21	1.99 ± 0.11
13	2.01 ± 0.11	2.03 ± 0.22	1.34 ± 0.05	1.41 ± 0.19	1.99 ± 0.15
14	4.14 ± 0.31	4.11 ± 0.42	1.78 ± 0.17	1.87 ± 0.31	4.26 ± 0.13
15	4.97 ± 0.16	5.10 ± 0.25	4.98 ± 0.19	5.18 ± 0.33	5.91 ± 0.13
16	4.00 ± 0.18	4.32 ± 0.19	4.02 ± 0.22	4.12 ± 0.28	8.51 ± 0.27
17	4.46 ± 0.23	4.43 ± 0.29	4.53 ± 0.16	4.53 ± 0.41	4.88 ± 0.32
18	1.50 ± 0.19	1.61 ± 0.20	1.42 ± 0.08	1.42 ± 0.11	1.68 ± 0.08
19	4.52 ± 0.32	4.71 ± 0.36	4.14 ± 0.34	4.20 ± 0.40	4.30 ± 0.17
20	4.44 ± 0.32	4.77 ± 0.34	4.60 ± 0.22	4.63 ± 0.40	3.70 ± 0.10
**d**					
**MARKER**	**B-GROUP**	**A-GROUP**	**B-GROUP**	**A-GROUP**	**B-GROUP**
	**VestS 8 (B)**	**VestS 9 (A)**	**VestS 9 (B)**	**VestS 10 (A)**	**VestS 10 (B)**
**ISR**	0.37 ± 0.05	0.43 ± 0.02	0.30 ± 0.09	0.41 ± 0.07	0.40 ± 0.06
1	0.54 ± 0.11	0.66 ± 0.07	0.94 ± 0.11	0.43 ± 0.13	0.51 ± 0.09
2	0.58 ± 0.12	0.63 ± 0.07	0.78 ± 0.17	0.64 ± 0.10	0.71 ± 0.14
3	0.63 ± 0.20	0.47 ± 0.07	0.69 ± 0.25	0.53 ± 0.13	0.57 ± 0.18
4	0.60 ± 0.14	0.90 ± 0.16	0.93 ± 0.15	0.98 ± 0.19	1.09 ± 0.16
5	1.01 ± 0.15	0.54 ± 0.10	0.81 ± 0.19	0.84 ± 0.12	1.00 ± 0.17
6	0.93 ± 0.07	0.93 ± 0.12	1.00 ± 0.19	0.91 ± 0.12	0.94 ± 0.31
7	0.94 ± 0.10	0.91 ± 0.08	0.99 ± 0.16	1.59 ± 0.27	1.71 ± 0.25
8	0.92 ± 0.15	0.93 ± 0.14	1.07 ± 0.16	1.00 ± 0.11	1.20 ± 0.13
9	0.89 ± 0.17	1.49 ± 0.08	1.54 ± 0.11	1.34 ± 0.13	1.52 ± 0.20
10	1.53 ± 0.18	1.33 ± 0.14	1.38 ± 0.13	0.97 ± 0.09	1.06 ± 0.14
11	1.68 ± 0.18	0.84 ± 0.07	0.87 ± 0.11	1.04 ± 0.17	1.26 ± 0.15
12	2.00 ± 0.21	0.96 ± 0.10	1.06 ± 0.18	1.01 ± 0.15	1.14 ± 0.19
13	2.00 ± 0.15	1.09 ± 0.15	1.12 ± 0.23	2.02 ± 0.16	2.18 ± 0.23
14	4.33 ± 0.33	2.10 ± 0.20	2.14 ± 0.23	4.03 ± 0.40	4.30 ± 0.49
15	5.84 ± 0.28	3.14 ± 0.26	3.22 ± 0.33	5.23 ± 0.39	4.78 ± 0.40
16	8.70 ± 0.35	2.98 ± 0.14	3.08 ± 0.29	5.16 ± 0.70	5.29 ± 0.21
17	4.92 ± 0.35	1.88 ± 0.12	1.94 ± 0.15	4.64 ± 0.30	4.76 ± 0.25
18	1.89 ± 0.20	1.10 ± 0.10	1.12 ± 0.14	1.21 ± 0.08	1.24 ± 0.13
19	4.76 ± 0.28	1.53 ± 0.51	2.01 ± 0.20	3.18 ± 0.37	3.32 ± 0.28
20	3.91± 0.15	1.80 ± 0.13	1.93 ± 0.13	2.41 ± 0.24	2.51 ± 0.28

*MaxS* Maxillary splint, *ISR* Internal split pin referencing (mean of *n* = 3 referencing), *VestS* Vestibular splint

**Fig. 3:**
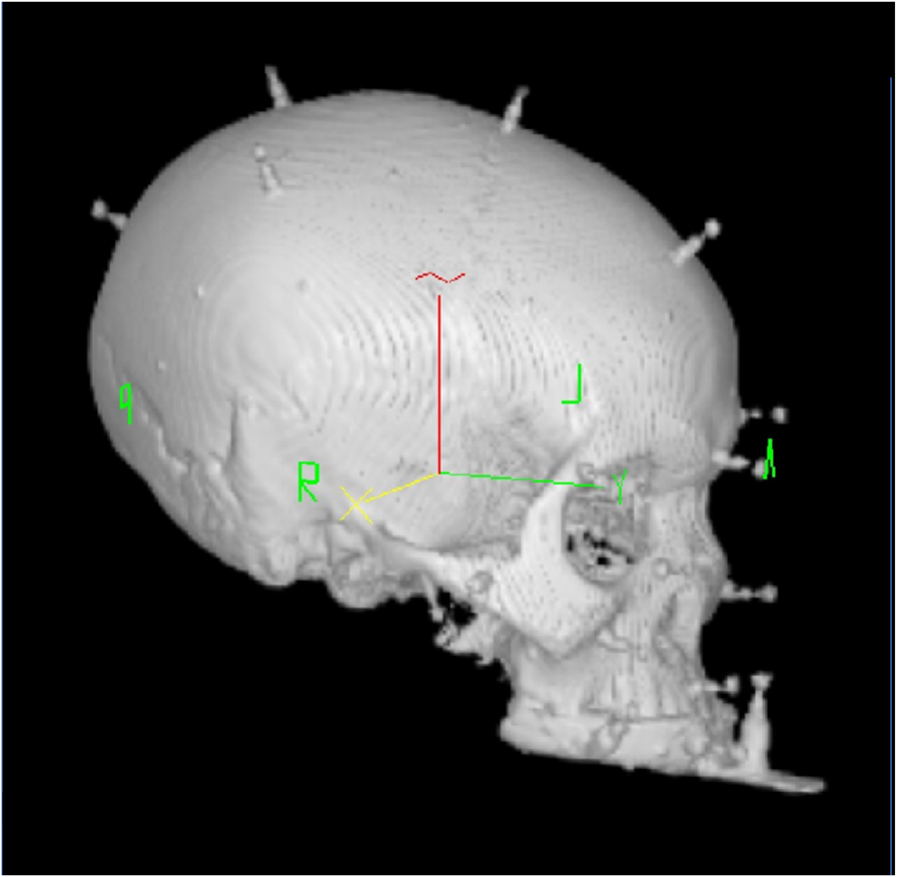
Three-dimensional reconstructed computed tomography of the skull with an inserted maxillary splint and twenty additionally inserted bone markers for metric accuracy testing

The skull was prepared with bone markers and a splint and then fastened to a head mounting plate made of foam material. The CT preformation was in an axial direction from caudal to cranial (gantry tilt 0°). The desk feed amounted to 2 mm, the slice thickness, and the reconstruction interval to 1 mm. After this, the prepared, radiologically examined skull and frame-fixed (e.g., Mayfield clamp) skull was positioned precisely 2 m from the infrared localisation camera (Fig. 3).

We used the Surgical Tool Navigator System with the software STP4 (Carl Zeiss, Jena, Germany) for three-dimensional navigation. The localisation of the determined point in the three space levels checked by way of an infrared pointer. A dynamic reference frame (DRF) firmly fastened to the Mayfield clamp with an adapter sent the space coordinates to an infrared beam camera. The localisation camera stands on a tripod above the area; the point appeared as a virtual point on the computer display.

**Fig. 4:**
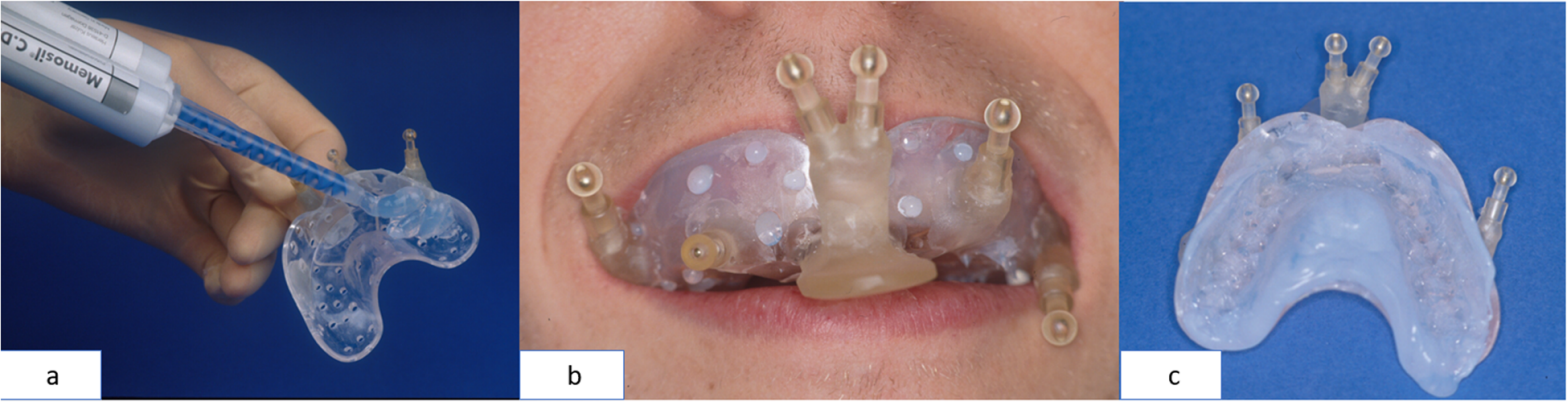
Production of an oral splint for the maxilla (maxillary splint, MaxS): **a** ready-made impression spoon armed with fiducial markers filled with a-silicone, (**b**) intraoral view of the MaxS, (**c**) extraoral view of MaxS

**Fig. 5:**
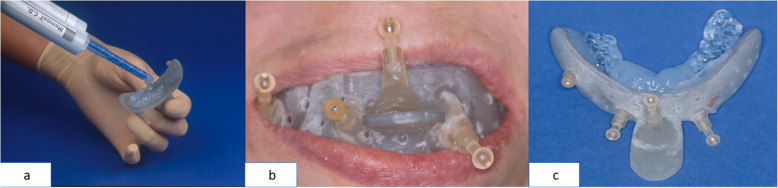
Production of an oral splint for edentulous patients (vestibular splint, VestS): **a** vestibular pacifier with fiducial markers is filled with a-silicone, (**b**) intraoral view of VestS, (**c**) extraoral view of VestS

The tripod's location and the head's adjustment in the Mayfield clamp concerning the beam camera did not change. The examination of the correct patient position on the monitor took place via the landmark test [20, 21]. By 400%, augmentation on the monitor in an enlarged multiplanar picture, control of the bone markers' position, and the splint fiducials in the axial view of the imported CT data was visible (Fig. 3). With the CT help, we compared three correlations, means ± standard deviations (SD). We checked the accuracy of five MaxS and five VestS as a function of the distance from the splint to all twenty bone markers. We used the average values of nine independent correlations per bone marker for each type of splint. We removed and reattached the splint ten times and repeated the measurements. Values determined in this way, we compared the bone markers' values and the splint fiducials' distance to the bone markers. We measured four fiducials for different splint types (Figs. 4 and 5) and fixed every splint to the upper jaw (reduced teeth).

We made a CT scan of a phantom head with a complete set of teeth and inserted oral splints. A correlation between markers *in vitro* (native skull) and the radiographic identified markers (Skull CT) showed the dependence of distance between the referenced bone markers.

### Statistical analysis

For statistical significance, we used the Kruskal-Wallis test and Tukey's test. A *p* value lower than 0.05 was considered statistically significant. We used various linear regression models and methods for determining correlation coefficients, such as Pearson's product correlation coefficient. The reference points' distance to the identical reference points in the reconstructed three-dimensional data record we called internal splint correlation (ISC). An examination of the correlation splint's correct position was possible utilising the landmark test. The accuracy calculation took place by comparisons of means for all twenty markers according to the parametric variance analysis (ANOVA). The Institute for Biometry, Department of Medical Biometry and Statistics at the Albert Ludwig University of Freiburg, Germany, provides the evaluation of the measurements.

## Results

For the investigation of the viscerocranium, the MaxS showed a deviation was 0.90 ± 0.27 mm (mean ± SD) with an ISC of 0.30, while the VestS showed a deviation of 0.90 ± 0.53 mm with an ISC of 0.30. The two splints were not statistically distinguishable regarding their exactness (Table 1). For the investigation of the splanchnocranium, the MaxS showed a deviation of 1.10 ± 0.39 mm with an ISC of 0.30, while the VestS showed a deviation of 1.20 ± 0.37 mm with an ISC of 0.30.

For the investigation of the neurocranium, the MaxS showed deviations of 1.45 ± 0.59 mm (ISC 0.30) mm, the accuracy for the VS was 1.70 ± 0.76 mm (ISC 0.30). As in other groups, the splints were not statistically distinguishable regarding their exactness (*p* < 0.048).

The accuracy for the palate area (nearby) was 0.71 ± 0.12 mm, the accuracy for the occipital bone (maximum distance) was 1.85 ± 0.46 mm.

The deviation was 1.28 ± 0.21 mm for the palatal area and 4.74 ± 0.21 mm for the occipital region.

**Fig. 6:**
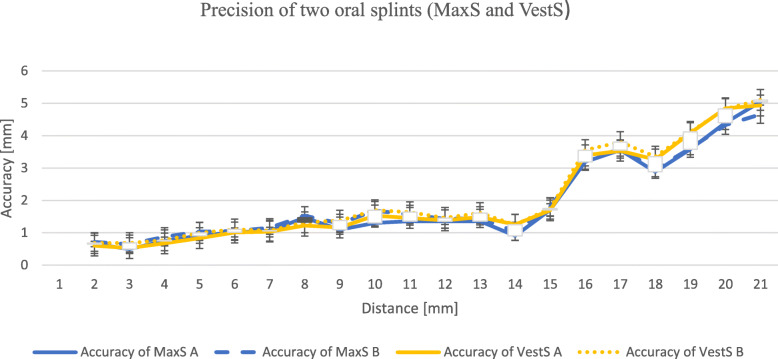
Accuracy of two oral splints for guided surgery immediately after fabrication and after ten attempts in relation to distance (mm). Data are given as mean ± standard deviation. *MaxS A* chairside fabricated maxillary splint, fixed on the dentate maxilla (Group A) immediately after fabrication. *MaxS B* chairside fabricated maxillary splint, fixed on the dentate maxilla (Group B) after ten attempts (removing and reattaching). *VestS A* chairside fabricated vestibular splint (pacifier), fixed as intermaxillary pacifier immediately after fabrication. *VestS B* chairside fabricated vestibular splint (pacifier), fixed as intermaxillary pacifier after ten attempts (removing and reattaching)

Differences in the accuracy of MaxS *versus* VestS did not become apparent (Fig. 6). We found an accuracy of less than 2 mm for both splints for a distance up to 15.2 cm, comparable to the linear distance of the splint focal point (subnasal) to the mastoid. VestS are held in place by soft tissues of the oral cavity. We tested on a dry skull, so soft tissue resilience was neglected.

## Discussion

Frameless navigation techniques can use different markers. Glued on skin markers, they show inaccuracies due to different skin resilience [3, 22]. The skin turgor, measured at preoperative investigation, is reduced in general anaesthesia due to the used medication. Wang et al. [21] recommend marking the skin with coloured pens to check the position or sticking back lost skin markers and attach extra skin markers. Maciunias et al. [6] reported the most exact marker with a deviation of 1.86 mm [23], 2.7 mm [10], or up to 4 mm [6].

Laser-skin-surface-contour scanning is used primarily in this field and could not match bone-implanted fiducial marker registration accuracy. Navigated landmarks used in magnetic resonance imaging datasets showed inaccuracies of at least 6.2 mm [24]. Golfinos et al. [10] describe deviations of up to 5.6 mm [10] for CTs. Ledderhose et al. [7] found inaccuracies of up to 2.4 mm for the lateral skull base for laser surface scan, so preoperatively fixed markers are still golden standard (0.33 ± 0.26 mm) [7] and for some regions, more accurate compared with dental splints (0.55 ± 0.28 mm).

In the context of the investigation, a bone target’s precision (measured as the bone-to-bone distance by the navigational system) had values from 0.79 ± 0.21 (palate) mm to 1.56 ± 0.23 mm (occiput). In comparison to that, our investigations with ready-made navigation splints showed an accuracy of 0.40 ± 0.10 mm for the palatal area and 2.13 ± 0.13 mm for the occipital bone. Therefore, the accuracy of navigation splints depends on the proximity of the splint to the determined area. Our results are comparable to the accuracy reported by Furuse et al. [22].

Luebbers et al. [23] published adequate precision in regions beyond the mid-face only by combining a dental splint with two bone-implanted markers on the lateral orbital rim, a result confirmed by other authors [25–27]. Combining oral splints and augmented reality or artificial intelligence-assisted surgical intervention for better intraoperative management is still missing [15, 28, 29].

MaxS is useful for patients with natural teeth or implant-based fixed prosthetics. For the MaxS, four sizes are useful: the smallest one for a child's or a juvenile upper jaw and bigger sizes for adult jaws. Patients with vomiting problems or pathological modifications of the palate (*e.g.*, easily bleeding hemangiomas) should use VestS. The pacifiers can also be prepared in different sizes and customised with impression materials in patients with a dysmorphic central face.

The preparation of a navigation splint is immediately possible for acutely traumatised patients. Compared with screws or invasive markers, the therapist can already use computer-assisted three-dimensional navigation in the primary operation after initial diagnostics. It enables evaluation of the result with the postoperative situation avoiding the harm of screw implantation [30]. Chairside-made oral splints may also improve intraoperative management in combination with augmented reality [27, 28] or artificial intelligence-assisted surgical intervention [29]. In the context of augmented reality, oral splints allow simplified automatic matching of the radiological data with the extraoral patient's image in real time due to fiducial markers. In the future, with the help of semitransparent augmented reality glasses or transparent smart glasses, it could be possible to superimpose the skeletal in the operational area by the meaning of mixed reality [31]. It is possible to store A-silicones that are dimensionally stable for a minimum of 5 years. They allow a later time for control. In a single tooth loss, the use of MaxS is exact because of multiple tooth contacts or palatal support with reduced resilience in bone-supported median-line raphe. We used one single phantom head, so the VestS suitability for a bite registration is hard to check. A patient-oriented attempt can verify the clinical gain of the various navigation splints [30]. Intraoral three-dimensional scanning of MaxS allows for digital reproduction in a short time in case of splintloss. Invasive markers are usually removed after surgical intervention and thus not available for follow-up.

Prefabricated splint precision is dependent on the distance to the determined point; the greater the distance of the splint to the envisaged region, the greater the inaccuracy. For up to 150 mm, a precision of approximately less than 2 mm is considered to be clinically acceptable. For a further distance (*e.g.*, periorbital or temporal regions), additional techniques such as surface matching or fiducial markers with a maximum of 4 or 5 markers are also recommended [30]. Some authors advocate extensions to oral splint to minimise errors [13]. The use of dental splints or the combination of oral splints with other navigational methods could improve the accuracy and the validation of surgical interventions and would be even a simple instrument for quality management after radiotherapy or surgery.

In conclusion, our study showed that dental splints provide sufficient accuracy compared with bone markers for a distance up to 15 cm and may opt for higher precision in the field of perioral ENT and maxillofacial surgery.

## Data Availability

The datasets used or analysed during the current study are available from the corresponding author on reasonable request.

## Abbreviations

CT: Computed tomography
DRF: Dynamic reference frame
ISC: Internal splint correlation
MaxS: Maxillary splint
SD: Standard deviation
VestS: Vestibular splint
